# Supplementary material for: p38-mediated phosphorylation at T367 induces EZH2 cytoplasmic localization to promote breast cancer metastasis
Source: Nat Commun. 2018 Jul 18;9:2801. doi: 10.1038/s41467-018-05078-8 (PMC6051995; doi:10.1038/s41467-018-05078-8)

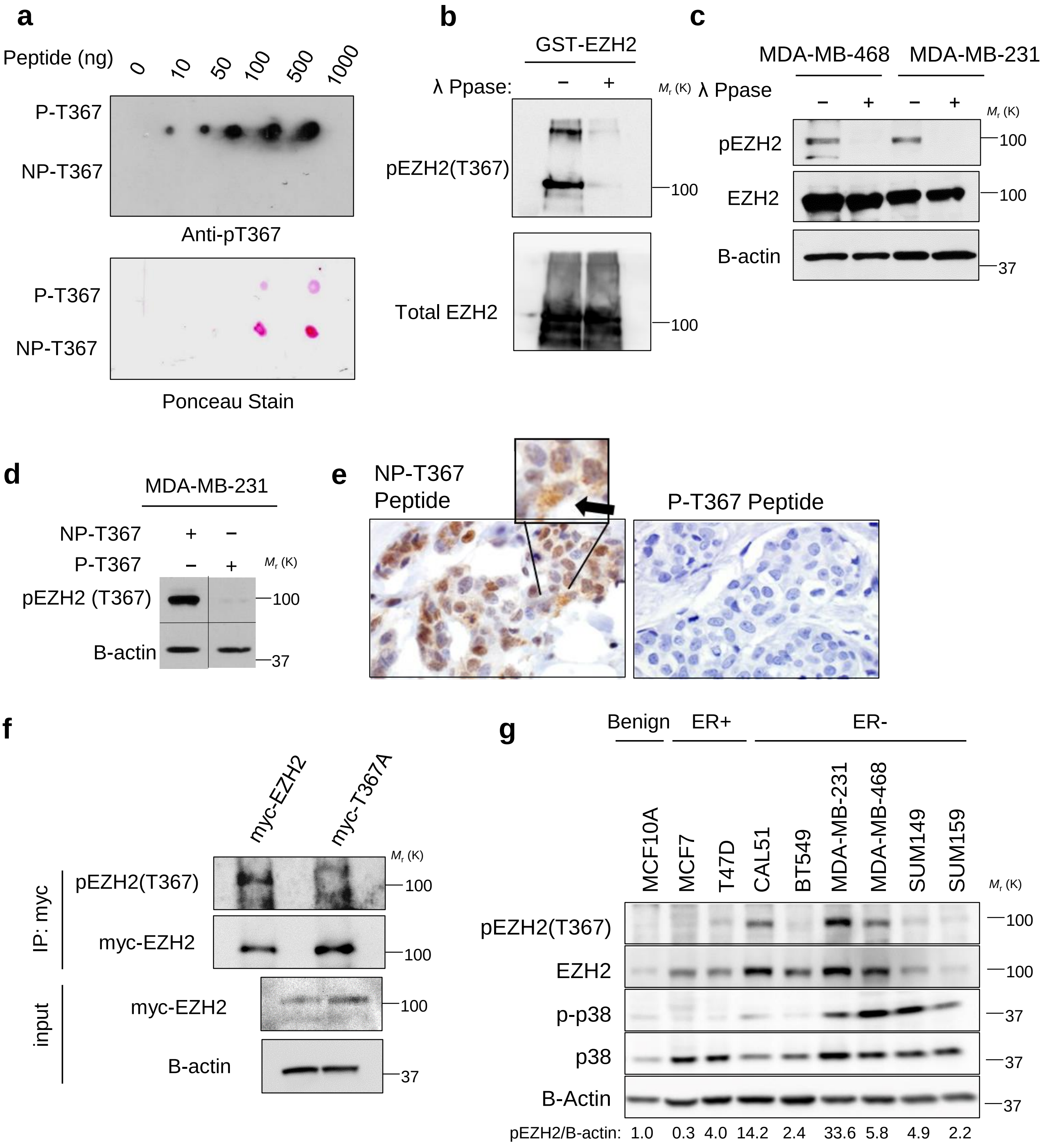

Supplementary Figure 1. pEZH2(T367) can be detected *in vitro* and *in vivo*. a Dot-blot assays performed using increasing quantities of phosphorylated (NSSRPS(pT)PTINVL) or non-phosphorylated (NSSRPSTPTINVL) peptides after incubation with affinity purified pEZH2 (T367) antibody. Ponceau stain shown as loading control. **b** Western blot of recombinant His-GST-EZH2 treated with lambda phosphatase to dephosphorylate protein. **c** Western blot using MDA-MB-468 and MDA-MB-231 whole cell lysates treated with lambda phosphatase to dephosphorylate protein. **d** Peptide competition western blot of MDA-MB-231 whole cell lysates using pEZH2 antibody pre-incubated with 200-fold molar excess of either non-phosphorylated or phosphorylated peptide.

e Peptide competition immunohistochemistry of an invasive breast carcinoma using pEZH2 antibody pre-incubated with 200-fold molar excess of either non-phosphorylated or phosphorylated peptide. **f** Immunoprecipitation of MDA-MB-231 cells transduced to express myc-WT-EZH2 or myc-T367A-EZH2 with myc antibody followed by western blot with pEZH2(T367) (left), with input (right). **g** Western blot analysis in a panel of cell lines with the indicated antibodies.

Variable	N	%
Cytoplasmic pEZH2(T367)		
Low	45	43.3
High	59	56.7
T-stage		
1	46	44.2
2	33	31.7
3	13	12.5
4	3	2.9
Missing	9	8.7
N Stage		
0	45	43.3
1	28	26.9
2	10	9.6
3	6	5.8
Missing	15	14.4
Histological Tumor Grade		
1	6	5.8
2	36	34.6
3	53	51.0
Missing	9	8.7
Estrogen Receptor		
Negative	40	38.5
Positive	51	49.0
Missing	13	12.5
Progesterone Receptor		
Negative	54	51.9
Positive	37	35.6
Missing	13	12.5
HER2/neu overexpression		
Negative	76	73.1
Positive	11	10.6
Missing	17	16.3

Supplementary Table 1. Clinical and pathological characteristics of the invasive carcinomas.

Variable	Cytoplasmic pEZH2(T367)				
	Low		High		
	n	%	n	%	p-value
T Stage					
1	23	53.5	24	45.3	0.47
2	15	34.9	18	34.0	
3-4	5	11.6	11	20.8	
N Stage					
0	17	46.0	28	53.8	0.69
1	12	32.4	16	30.8	
2-3	8	21.6	8	15.4	
Histological Tumor Grade					
1	5	12.5	1	1.8	0.028
2	18	45.0	18	32.7	
3	17	42.5	36	65.5	
Estrogen Receptor					
Negative	9	22.5	31	60.8	0.0003
Positive	31	77.5	20	39.2	
Progesterone Receptor					
Negative	15	37.5	39	76.5	0.0002
Positive	25	62.5	12	23.5	
HER2/neu overexpression					
Negative	37	92.5	39	83.0	0.18
Positive	3	7.5	8	17.0	
Triple Negative					
No	34	85.0	23	50.0	0.0006
Yes	6	15.0	23	50.0	

Supplementary Table 2. Associations between pEZH2(T367) cytoplasmic expression and clinical and pathological characteristics on the invasive carcinomas.

Epithelial origin	Cyto pEZH2
Lung (n)	
normal (1)	-
adenocarcinoma (5)	+
Intestine (n)	
colon (3)	-
adenocarcinoma (3)	+
Kidney (n)	
normal (2)	-
renal cell carcinoma (3)	+
Prostate (n)	
normal (1)	-
adenocarcinoma (2)	-
Liver (n)	
normal (3)	-
carcinoma (1)	+
Thyroid (n)	
normal (1)	-
carcinoma (5)	+

Non-epithelial origin	Cyto pEZH2
Brain (n)	
normal (2)	-
glioma (2)	-
Smooth muscle (n)	
normal (5)	-
leiosarcoma (1)	-
Testis (n)	
normal (1)	+
seminoma (1)	-
Lymphocytes (n)	
lymph node (2)	-
Hodgkin's lymphoma (3)	+
Thymus (n)	
normal (2)	-
thymoma (1)	-

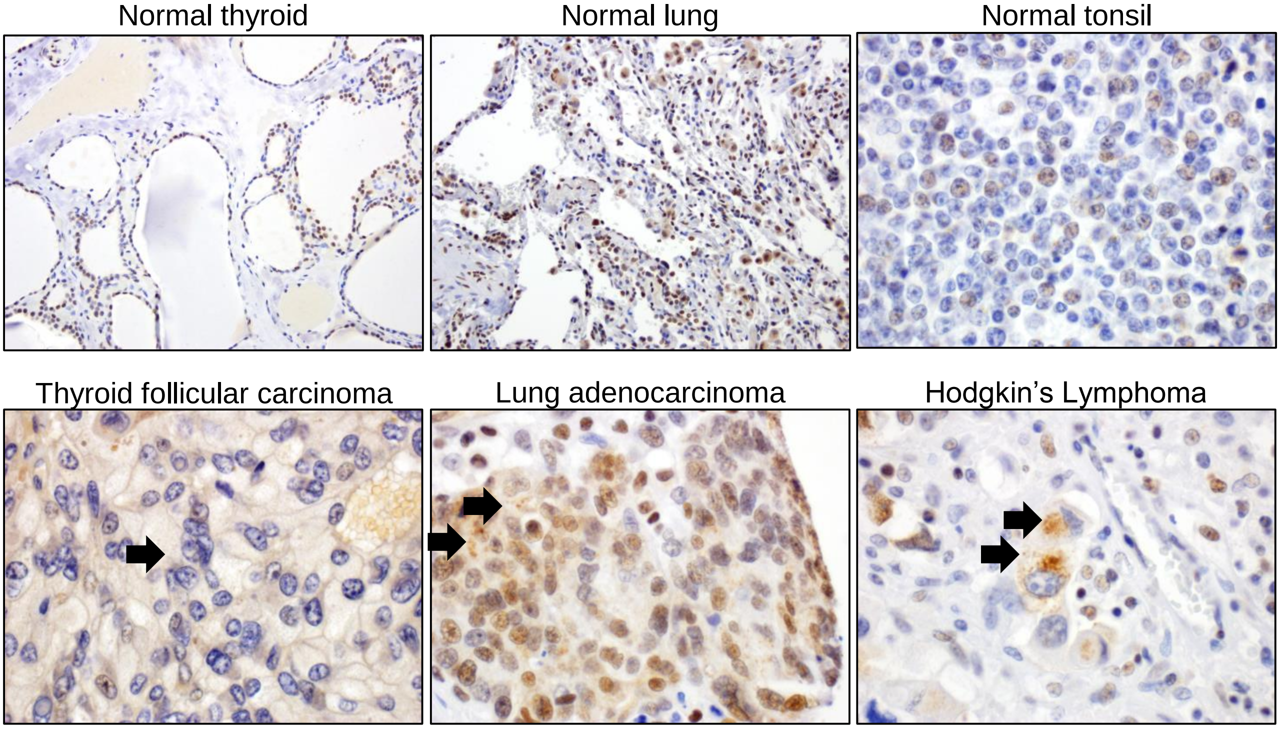

Supplementary Figure 2. Survey of pEZH2(T367) expression in normal and malignant tissue. (Table, top) pEZH2(T367) expression in normal and neoplastic tissues of epithelial (left) and non-epithelial (right) origin. The presence of cytoplasmic pEZH2(T367) tissue is noted. (Bottom) Example immunohistochemical images of pEZH2(T367) expression patterns in normal thyroid, lung, and tonsil, and thyroid follicular carcinoma, lung adenocarcinoma, and Hodgkin's lymphoma. Images taken at 400X magnification. Arrows delineate cells with pEZH2 in the cytoplasm.

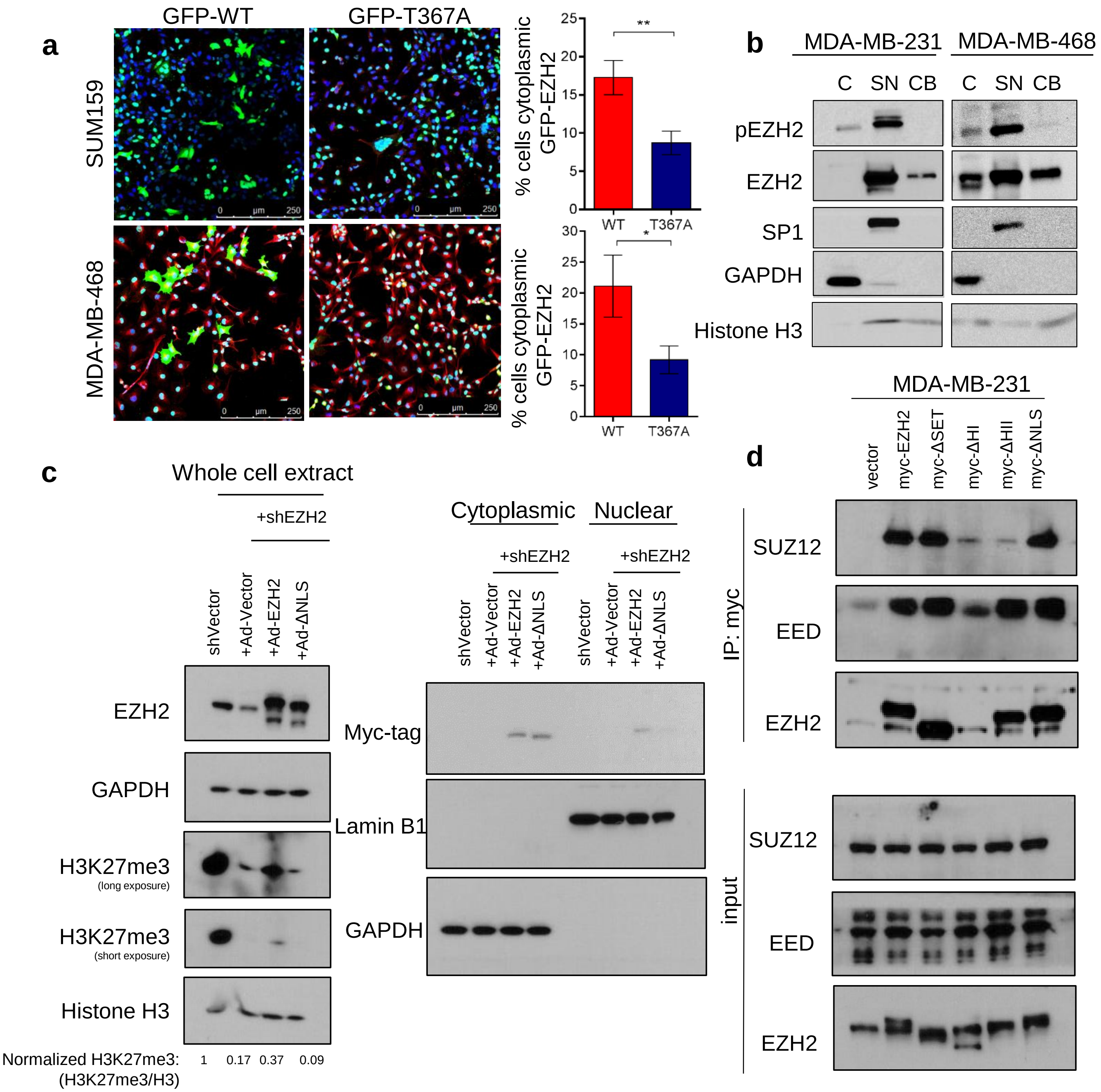

Supplementary Figure 3. pEZH2(T367) is associated with cytoplasmic localization. a Immunofluorescence images of SUM159 and MDA-MB-468 cells transduced with lentivirus to express GFP-EZH2 wild-type, or T367A protein. The number of non-mitotic cells expressing cytoplasmic EZH2 was quantified from ≥50 cells for each condition in three fields and graphicized on the right. Red, alpha-Tubulin, blue, DAPI. Scale bars, 250 um. Data are representative from a single experiment that was repeated at least three times, each with three technical replicates, and are presented as mean ± SD. **b** Western blot analysis of MDA-MB-231 and MDA-MB-468 cell lines subjected to fractionation into cytoplasmic (C), soluble nuclear (SN), and chromatin-bound (CB) fractions. SP1, GAPDH, and Histone H3 used as subcellular fractionation compartment controls.

c Western blot analysis of MDA-MB-231 transduced with lentivirus to knockdown EZH2 and rescue with Ad-EZH2 or Ad-ΔNLS EZH2 whole cell lysate (left) and fractionated cells (right). **d** Immunoprecipitation of myc-tag from MDA-MB-231 cells transduced with adenovirus to express vector, wild-type EZH2, or ΔSET, ΔHI, ΔHII, or ΔNLS domain deletion constructs. Western blot performed on immunoprecipitated protein for PRC2 members SUZ12 and EED. Bottom, input. *$p \leq 0.05$; **$p \leq 0.01$

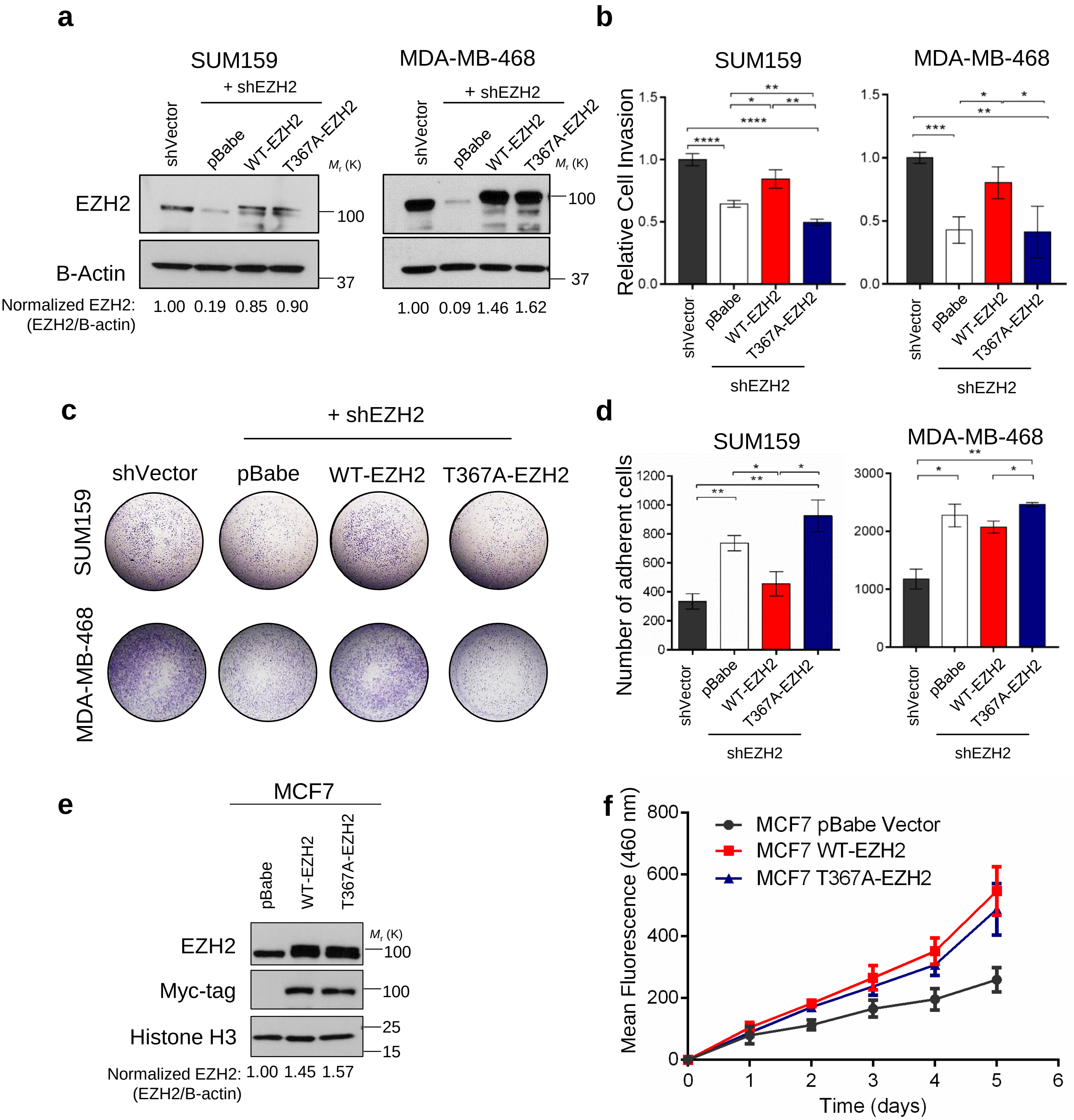

Supplementary Figure 4. pEZH2(T367) regulates migration, invasion, and adhesion of SUM159 and MDA-MB-68 breast cancer cells in *vitro*. a Western blot analysis of SUM159 and MDA-MB-468 breast cancer cells showing EZH2 knockdown after lentiviral transduction with control shRNA (shVector) or 3’ UTR EZH2-targeting shRNA (shEZH2) and rescue with pBabe vector, myc-tagged WT-EZH2, or T367A-EZH2.

b Cells described in (A) employed in a reconstituted Boyden basement membrane invasive chamber assay. **c** Representative chambers of (B) after crystal violet staining. All cells were counted using ImageJ. **d** Cells described in (A) employed in a cell attachment assay. **e** Western blot analysis of ER+ MCF7 cells transduced with lentivirus to express pBabe vector, WT-EZH2, or T367A-EZH2. **f** Cells described in E employed in a time course proliferation assay determined using Hoescht 33258 to quantify dsDNA. Functional data in this figure are from at least three independent biological replicates carried out with at least three technical replicates, and are presented as mean ± SD. $^{*}p \leq 0.05$; $^{**}p \leq 0.01$; $^{***}p \leq 0.005$; $^{****}p \leq 0.0001$

a

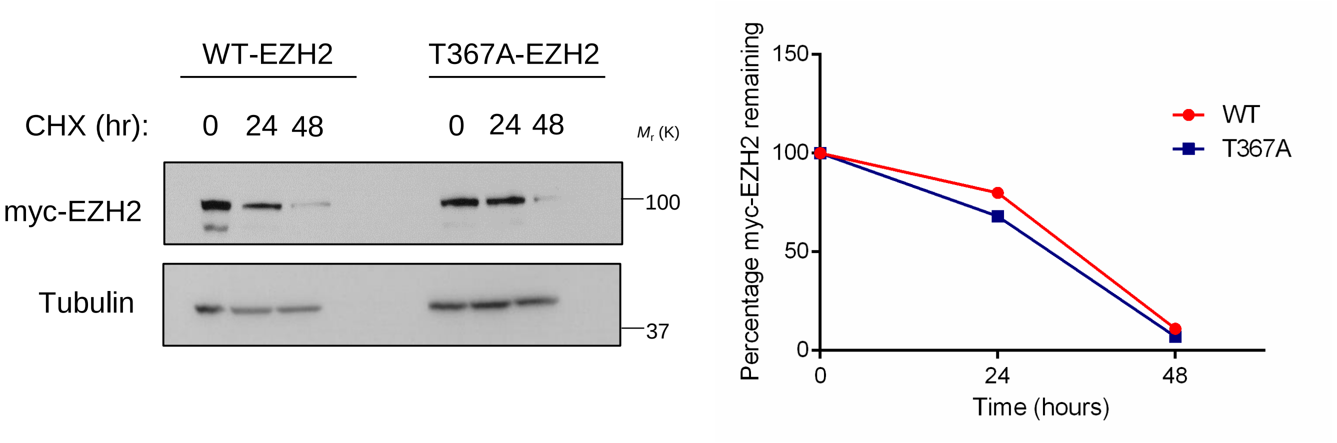

b

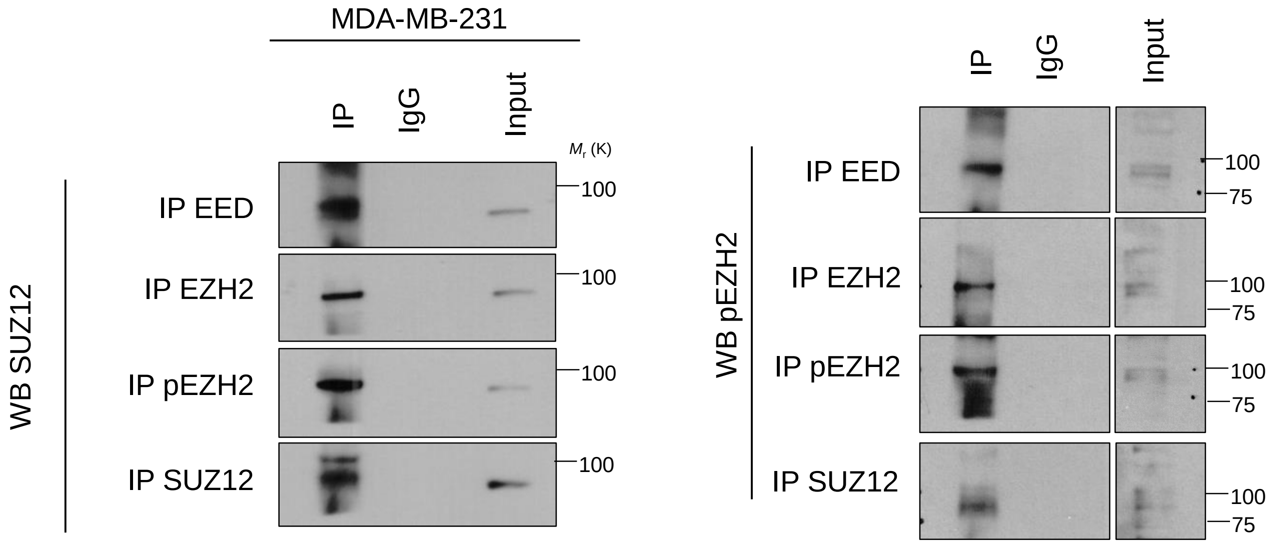

Supplementary Figure 5. T367A-EZH2 not associated with stability, and pEZH2 can bind PRC2 members SUZ12 and EED. a Cycloheximide (CHX) pulse chase assay in MDA-MB-231 cells transduced with lentivirus to express myc-WT-EZH2 or myc-T367A-EZH2. Cells were seeded in a 6-well plate and treated with 100ug/ml CHX for 0, 24, and 48 hours. Left, immunoblot of myc-tag. Right, plotted quantified immunoblot, with myc-EZH2 stability over time normalized to tubulin expression. **b** Co-immunoprecipitation followed by western blot experiments using the indicated antibodies from MDA-MB-231 whole cell lysates showing interaction of pEZH2 with SUZ12 and EED.

WT-EZH2 and T367A-EZH2 known interactors

Gene ID	WT FC-A	T367A FC-A	WT Normalized FC-A	T367A Normalized FC-A	Average WT SC	Average T367A SC	Average Control SC	WT-SP	T367A-SP
EZH2	71.2	270.9	1.0000	1.0000	92.7	338.7	0.0	1.0	1.0
SUZ12	39.3	106.1	0.5519	0.3918	52.0	131.7	0.0	1.0	1.0
EED	38.2	224.8	0.5363	0.8297	49.3	298.0	0.0	1.0	1.0
PHF19	4.8	17.0	0.0670	0.0628	5.0	20.0	0.0	1.0	1.0
PHF1	4.4	18.1	0.0624	0.0666	4.3	20.3	0.0	1.0	1.0
JARID2	3.9	10.4	0.0553	0.0385	4.0	11.0	0.0	1.0	1.0
TK1	3.0	4.8	0.0426	0.0179	2.7	4.7	0.0	0.7	1.0
CTNNB1	2.7	3.2	0.0375	0.0117	11.0	12.3	2.3	0.5	0.6
PHB2	2.4	1.4	0.0337	0.0050	11.0	4.3	3.7	0.3	0.0
RASA1	2.3	2.1	0.0320	0.0076	1.3	1.0	0.0	0.3	0.3
SMS	2.2	1.4	0.0302	0.0050	1.3	0.3	0.0	0.6	0.0
USP7	1.5	2.0	0.0212	0.0072	9.7	13.3	4.3	0.1	0.3
GNAS	1.4	1.2	0.0195	0.0044	4.0	3.3	1.7	0.3	0.3
WDR61	1.4	2.3	0.0190	0.0085	0.3	1.3	0.0	0.0	0.3
RBBP4	1.4	2.3	0.0190	0.0083	20.7	32.7	10.3	0.1	0.7
CDK1	1.3	1.6	0.0178	0.0058	8.3	10.7	4.7	0.1	0.4
RELA	0.7	1.4	0.0097	0.0052	1.0	3.3	1.3	0.0	0.5
STAT3	0.7	1.7	0.0095	0.0063	5.3	13.3	5.0	0.0	0.4
MTF2	0.0	3.8	0.0000	0.0139	0.0	3.7	0.0	0.0	0.7

Supplementary Table 3. WT- and T367A-EZH2 known interactors identified by mass spectrometry. List of differential interactors identified from actin-binding set with fold-change A (FC-A) scores and normalized FC-A scores based on total EZH2 pulldown. Average WT- and T367A spectral counts (SC) and SAINT probabilities (SP) are also displayed.

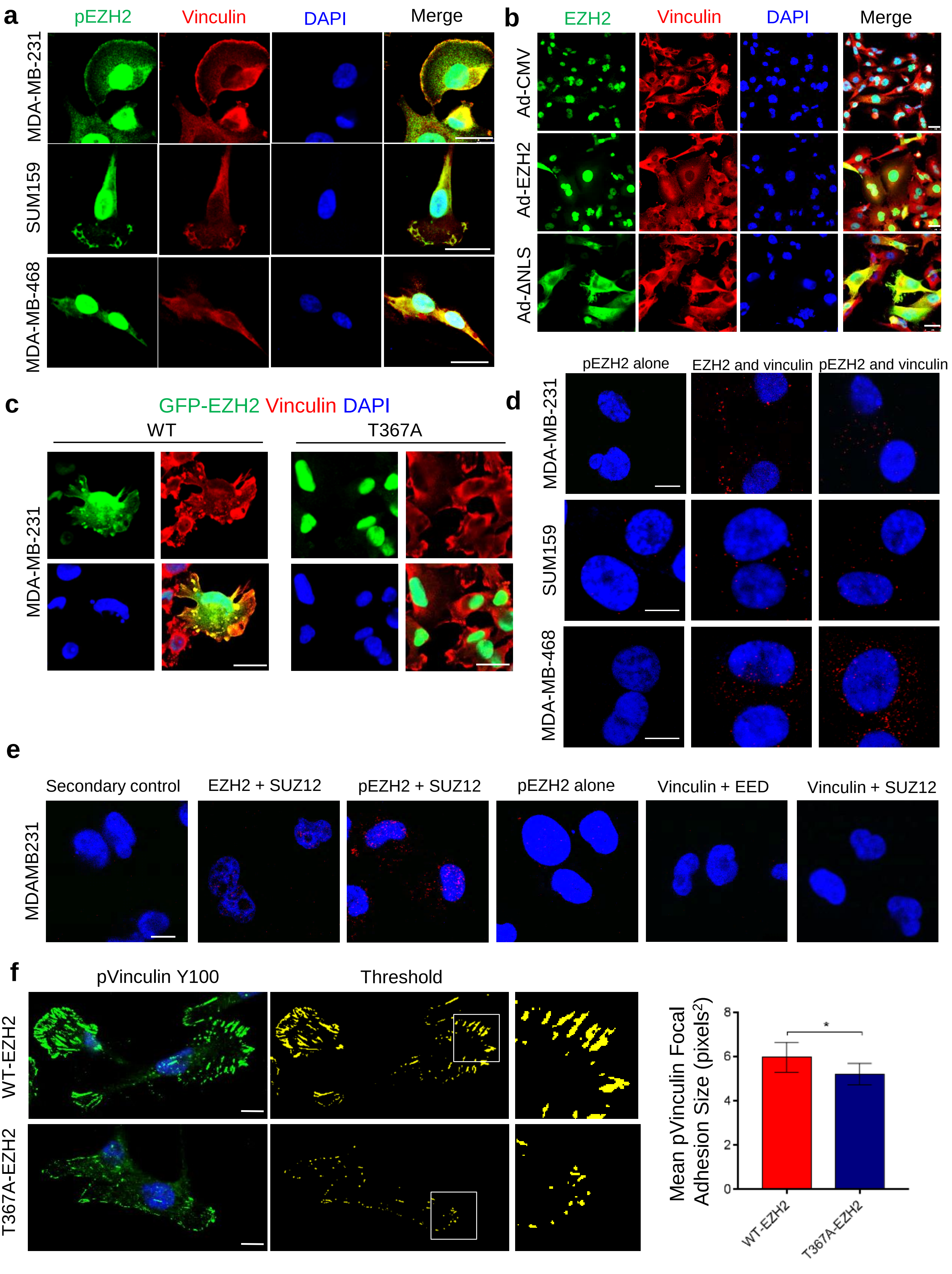
a
pEZH2
Vinculin
DAPI
Merge
MDA-MB-231
SUM159
MDA-MB-468
b
EZH2
Vinculin
DAPI
Merge
Ad-CMV
Ad-EZH2
Ad-ΔNLS
c
GFP-EZH2 Vinculin DAPI
WT
T367A
MDA-MB-231
d
pEZH2 alone
EZH2 and vinculin
pEZH2 and vinculin
MDA-MB-231
SUM159
MDA-MB-468
e
Secondary control
EZH2 + SUZ12
pEZH2 + SUZ12
pEZH2 alone
Vinculin + EED
Vinculin + SUZ12
MDAMB231
f
pVinculin Y100
Threshold
WT-EZH2
T367A-EZH2
Mean pVinculin Focal Adhesion Size (pixels^2)
0
2
4
6
8
*
WT-EZH2
T367A-EZH2

Supplementary Figure 6. Co-localization of EZH2 and vinculin **in breast cancer cells. a** Immunofluorescence staining of pEZH2(T367) and vinculin counterstained with DAPI imaged by confocal microscopy in the indicated breast cancer cell lines. Scale bars, 25 um. **b** Immunofluorescence imaging of MDA-MB-231 cells transduced with Ad-vector, Ad-EZH2, or Ad-ΔNLS mutant. Cells were stained for EZH2 (green) and vinculin (red), and counterstained with DAPI. Scale bars, 25 uM. **c** Immunofluorescence images of MDA-MB-231 cells transduced with GFP-WT-EZH2 or GFP-T367A-EZH2 (green); vinculin (red). Scale bars, 25 um. **d-e** Additional proximity ligation assay images in the indicated cell lines. Scale bars, 10 um . **e** Additional images from proximity ligation experiments corresponding to Fig.7a. **f** Immunofluorescence imaging of MDA-MB-231 knockdown-rescue cells expressing WT-EZH2 or T367-EZH2 and seeded on fibronectin stained with pVinculin(Y100) (left), with example threshold image used for quantitation of focal adhesion size. Representative data from an experiment quantified (right) are displayed as mean ± SD. Experiments were repeated three times with at least three technical replicates. $*p \leq 0.05$

Supplementary Table 4. Sequencing and Mutagenesis Primers

Primer Name	Sequence
**EZH2_REV1_400_1**	TTCTTCAATGAAAGTACCATCCTG
**EZH2_REV2_800_401**	TGAAAGGAGTGTAAGCTTTGCT
**EZH2_REV3_1200_801**	TCAGAGGAGCTCGAAGTTTCA
**EZH2_REV4_1500_1201**	TTCAGCTGTATCTTTCTGCAGTG
**EZH2_REV5_2000_1601**	TGCATCCACCACAAAATCAT
**EZH2_FWD1_401_800**	GCAGAATTTTATGGTGGAAGATG
**EZH2_FWD2_801_1200**	AGGCGCACTTCCTCCTGAAT
**EZH2_FWD3_1201_1600**	TAGGGAAGCAGGGACTGAAA
**EZH2_FWD4_1601_2000**	CCATGTTTACAACTATCAACCCTG
**EZH2_FWD5_2001_2256**	GATGAAGCTGACAGAAGAGGG
**pBABE backbone 3'**	ACCCTAACTGACACACATTCC (per Addgene, Weinberg Lab)
**pBABE backbone 5'**	CTTTATCCAGCCCTCAC (per Addgene, Weinberg Lab)
**EZH2 T367A FWD**	ATTAATGGTGGGGGCGCTGGGCCTGCTAC
**EZH2 T367A REV**	GTAGCAGGCCCAGCGCCCCCACCATTAAT

Supplementary Figure 7. Raw/uncropped western blot scans

Figures 2b-2c

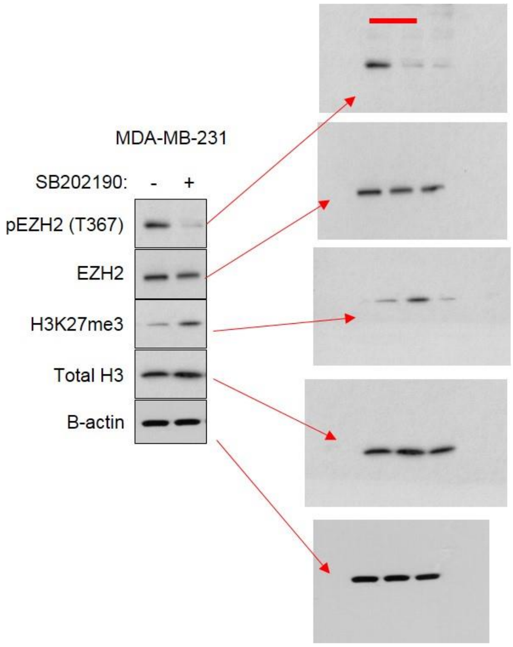

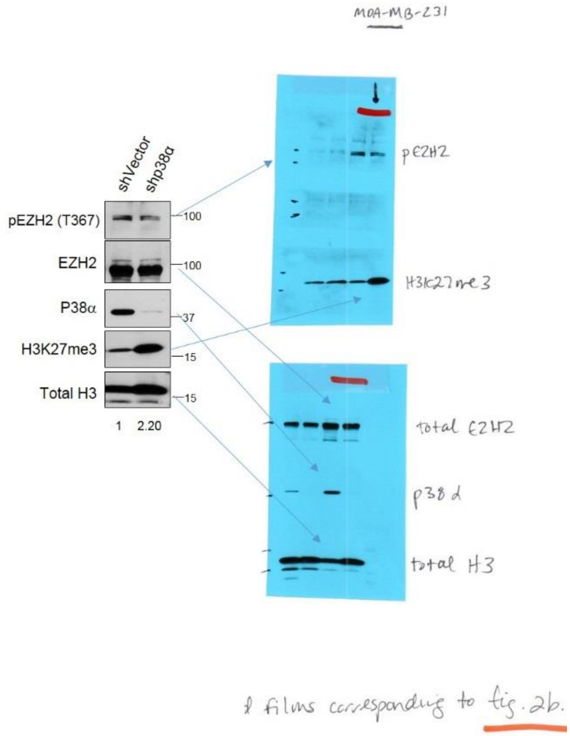

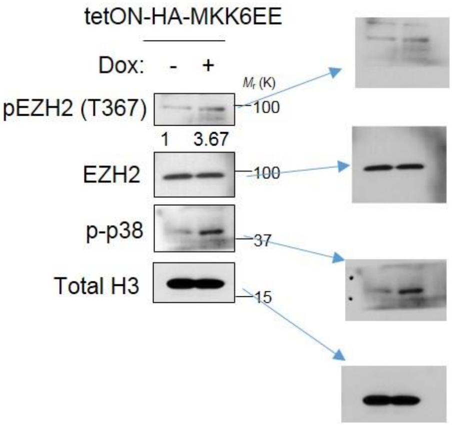

Supplementary Figure 7. Raw/uncropped western blot scans

Figure 3d

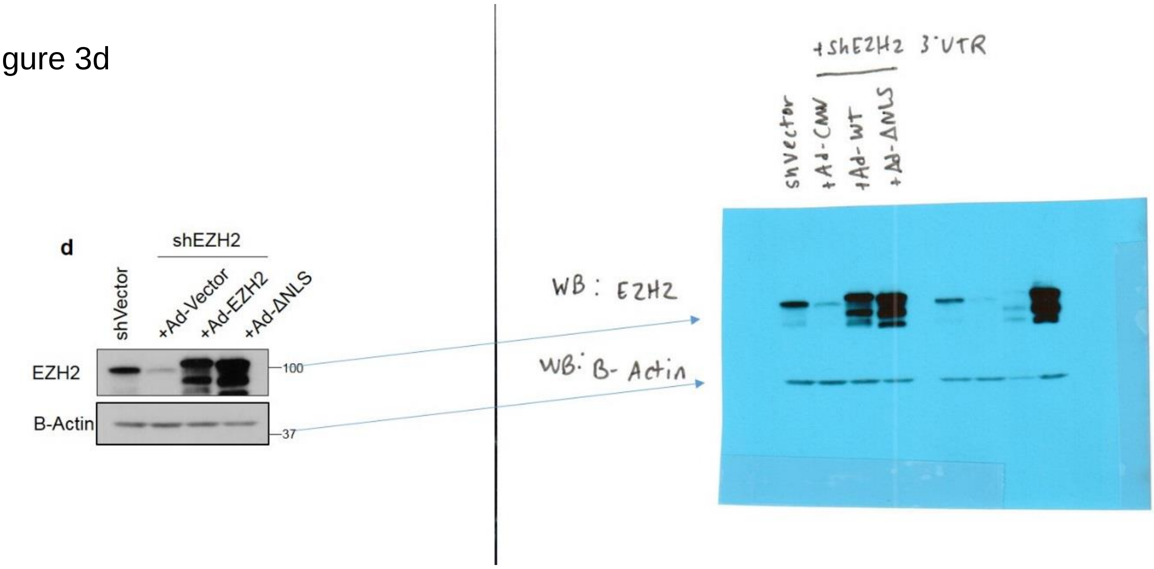

Figure 4a

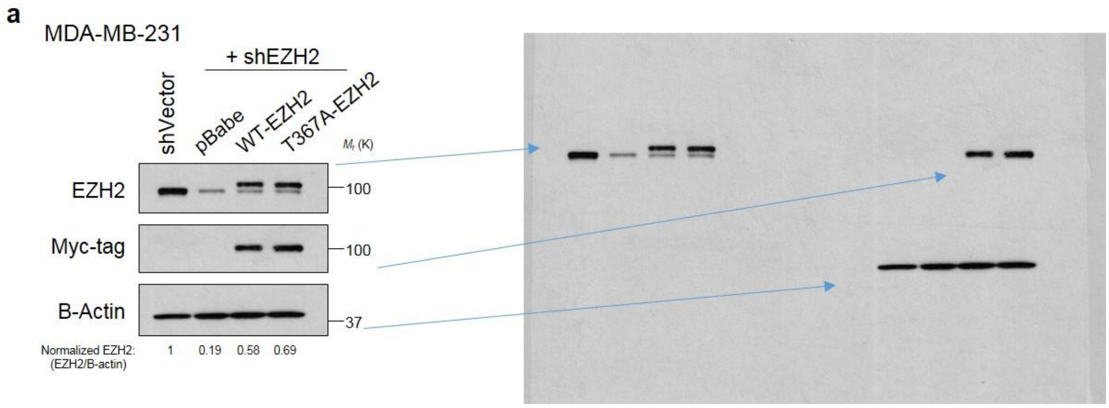

Supplementary Figure 7. Raw/uncropped western blot scans

Figures 7c-e

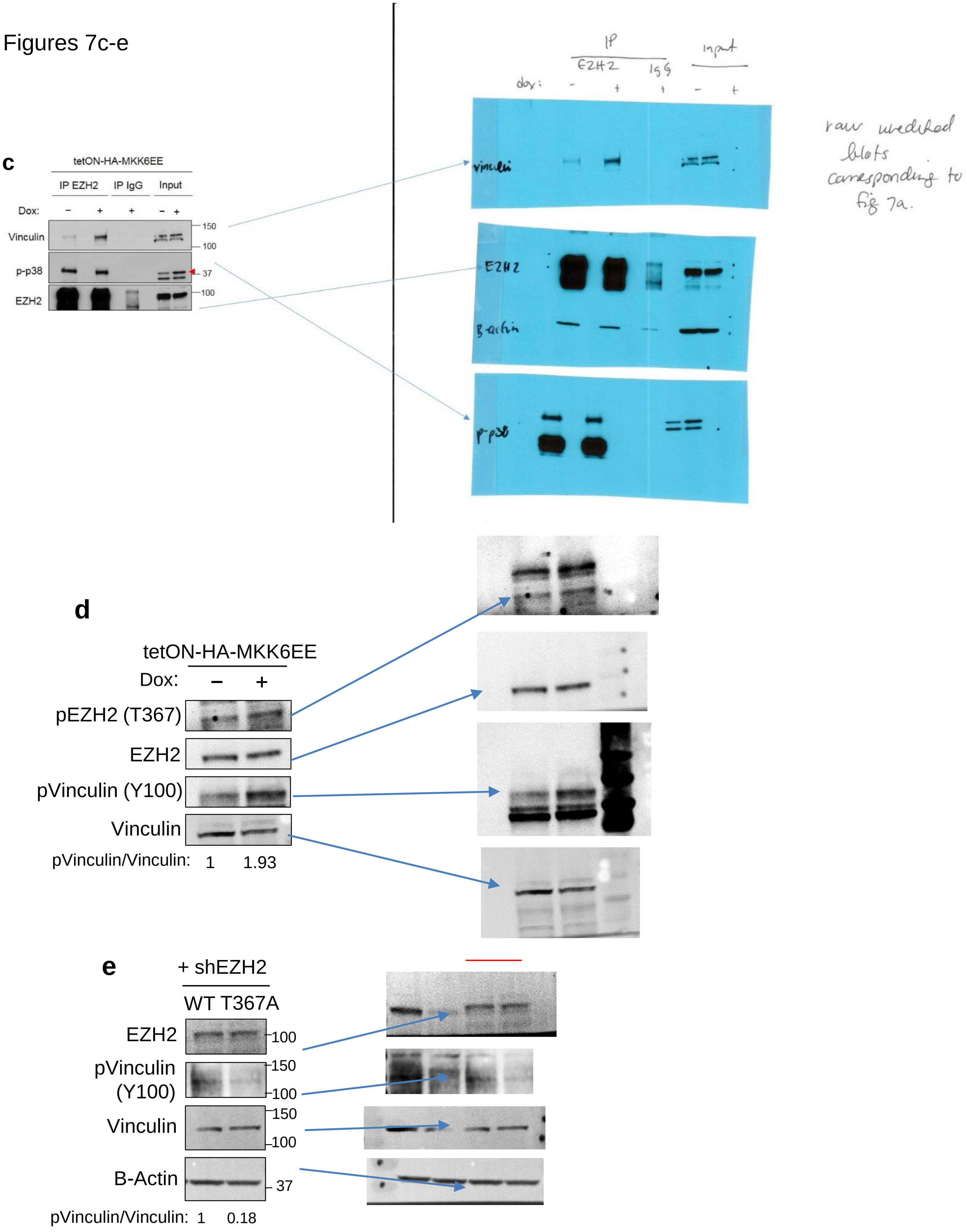

Supplementary Figure 7. Raw/uncropped western blot scans

Supplementary Figure 1a-d

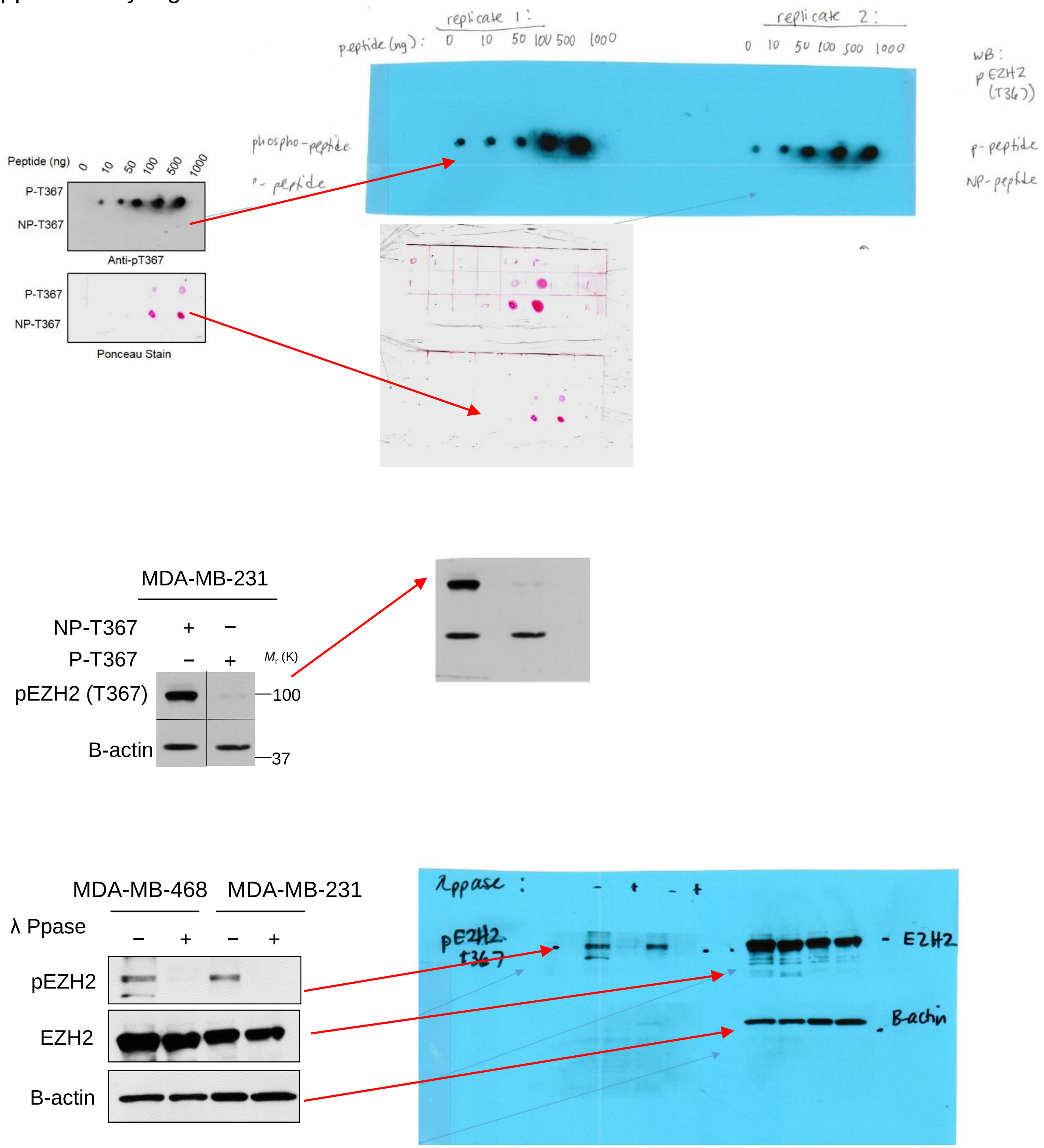

Supplementary Figure 7. Raw/uncropped western blot scans

Supplementary Figure 1g

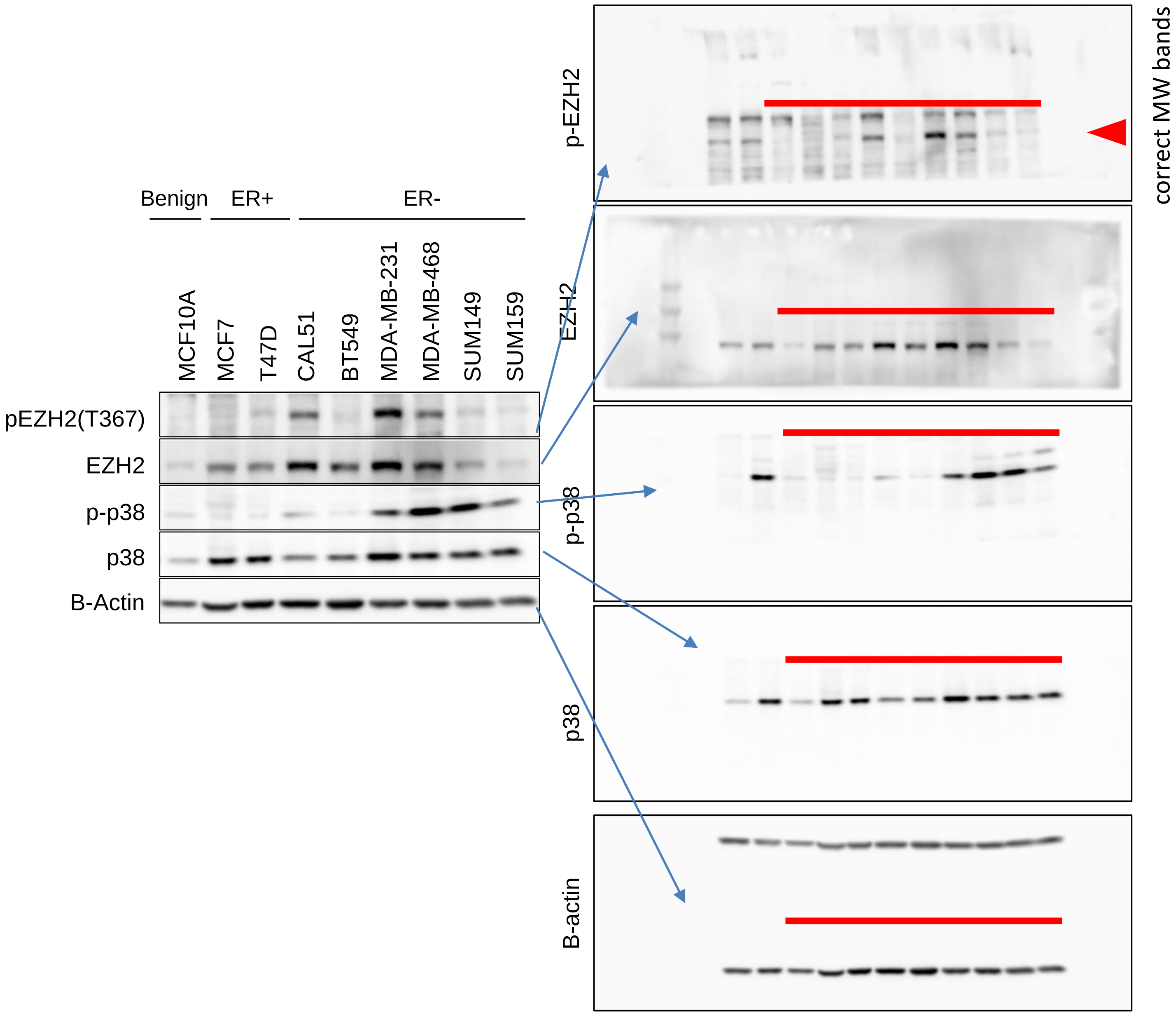

Supplementary Figure 7. Raw/uncropped western blot scans

Supplementary Figure 3b

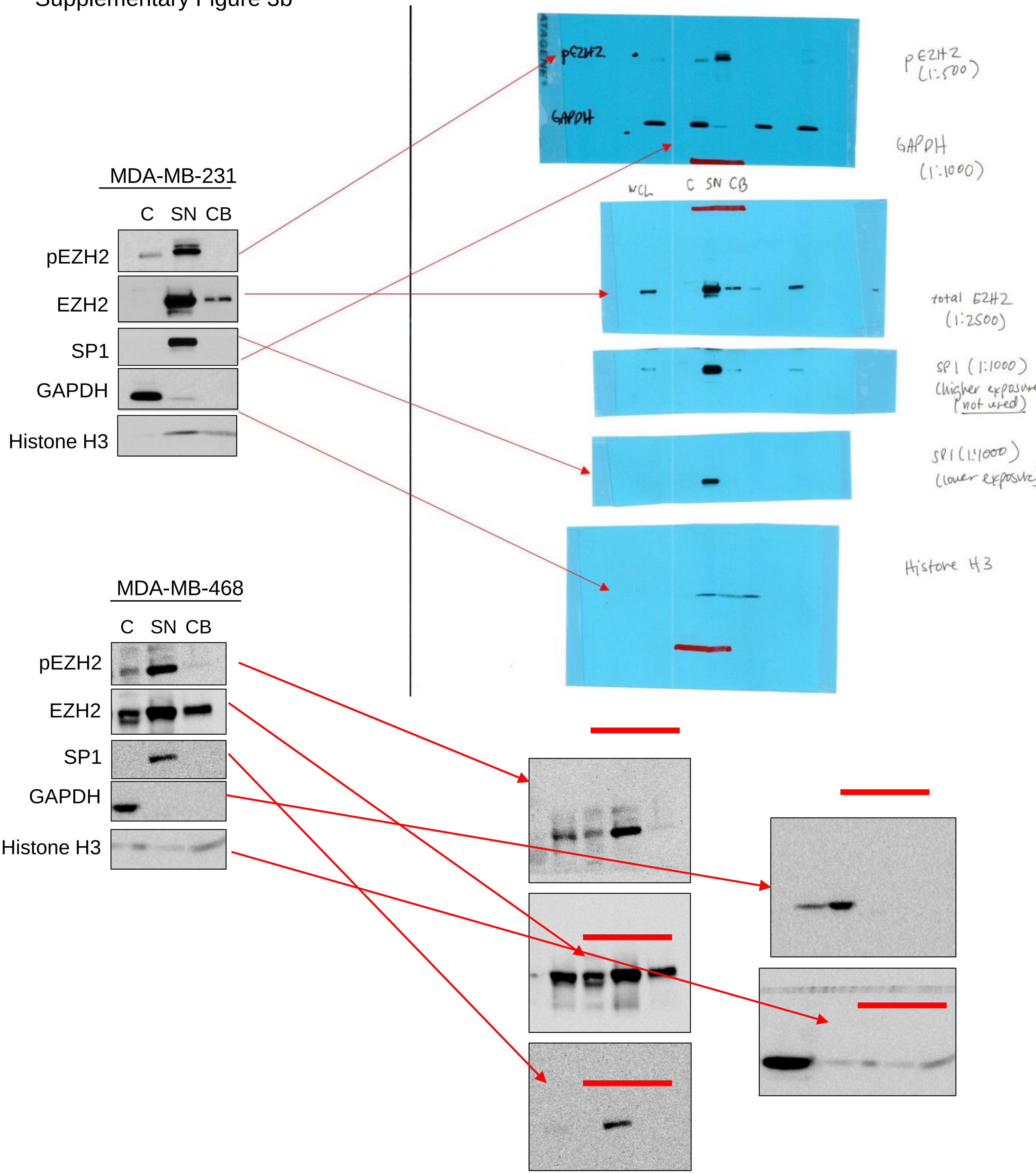

Supplementary Figure 7. Raw/uncropped western blot scans

Supplementary Figure 4a

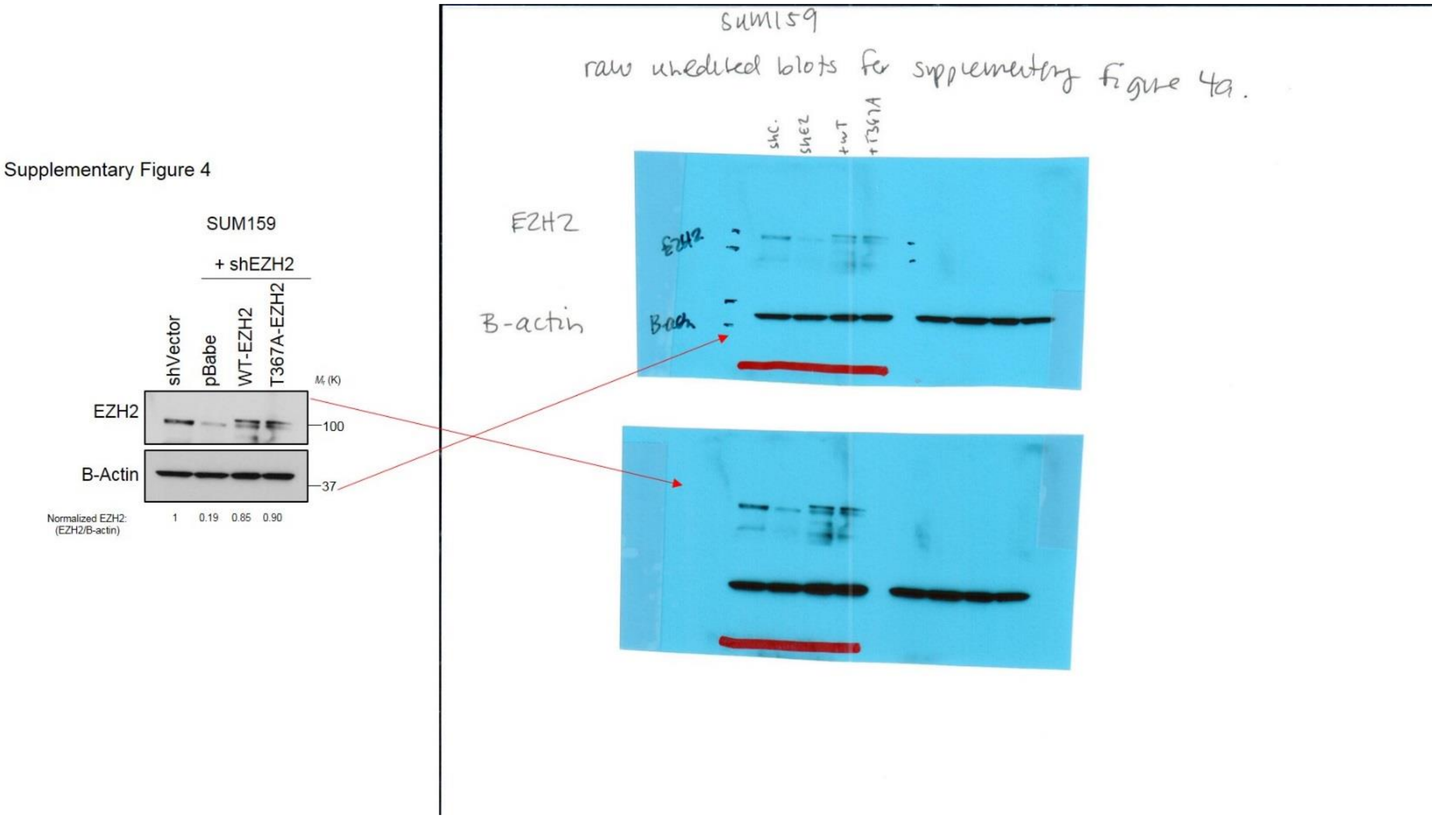

Supplementary Figure 4e

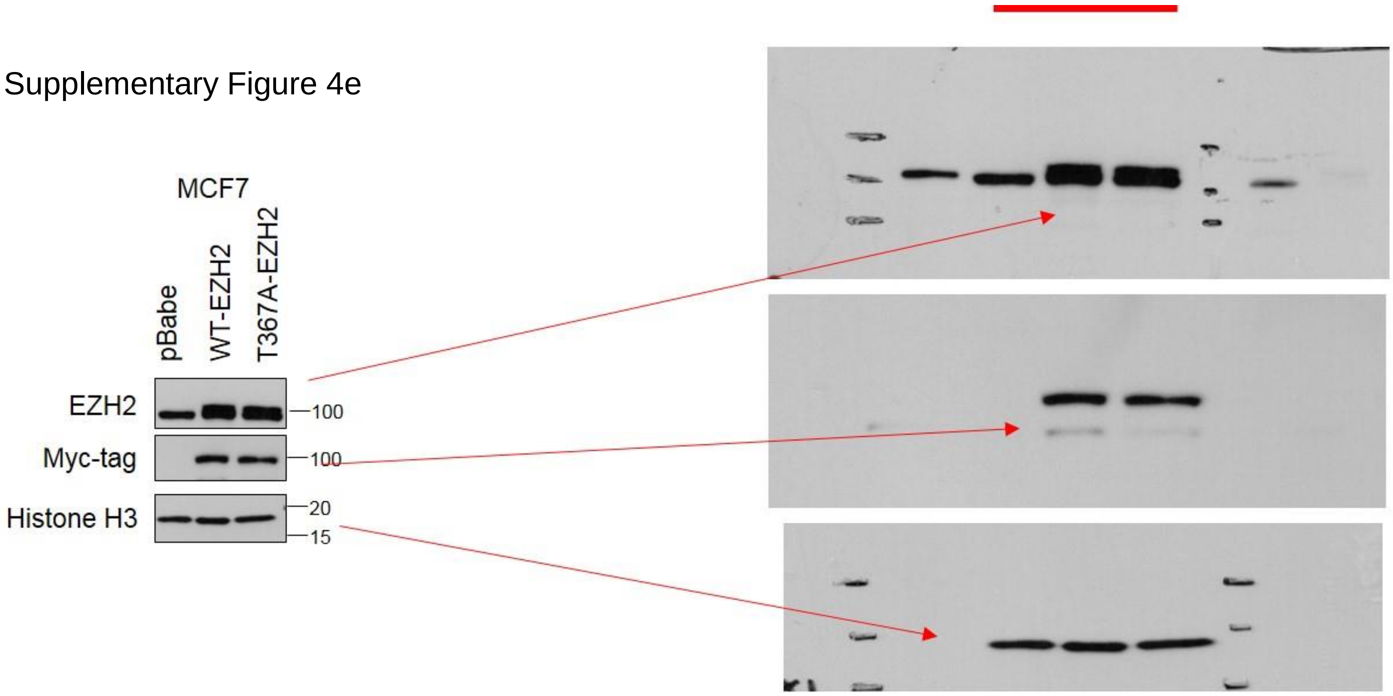

Supplementary Figure 7. Raw/uncropped western blot scans

Supplementary Figure 5a

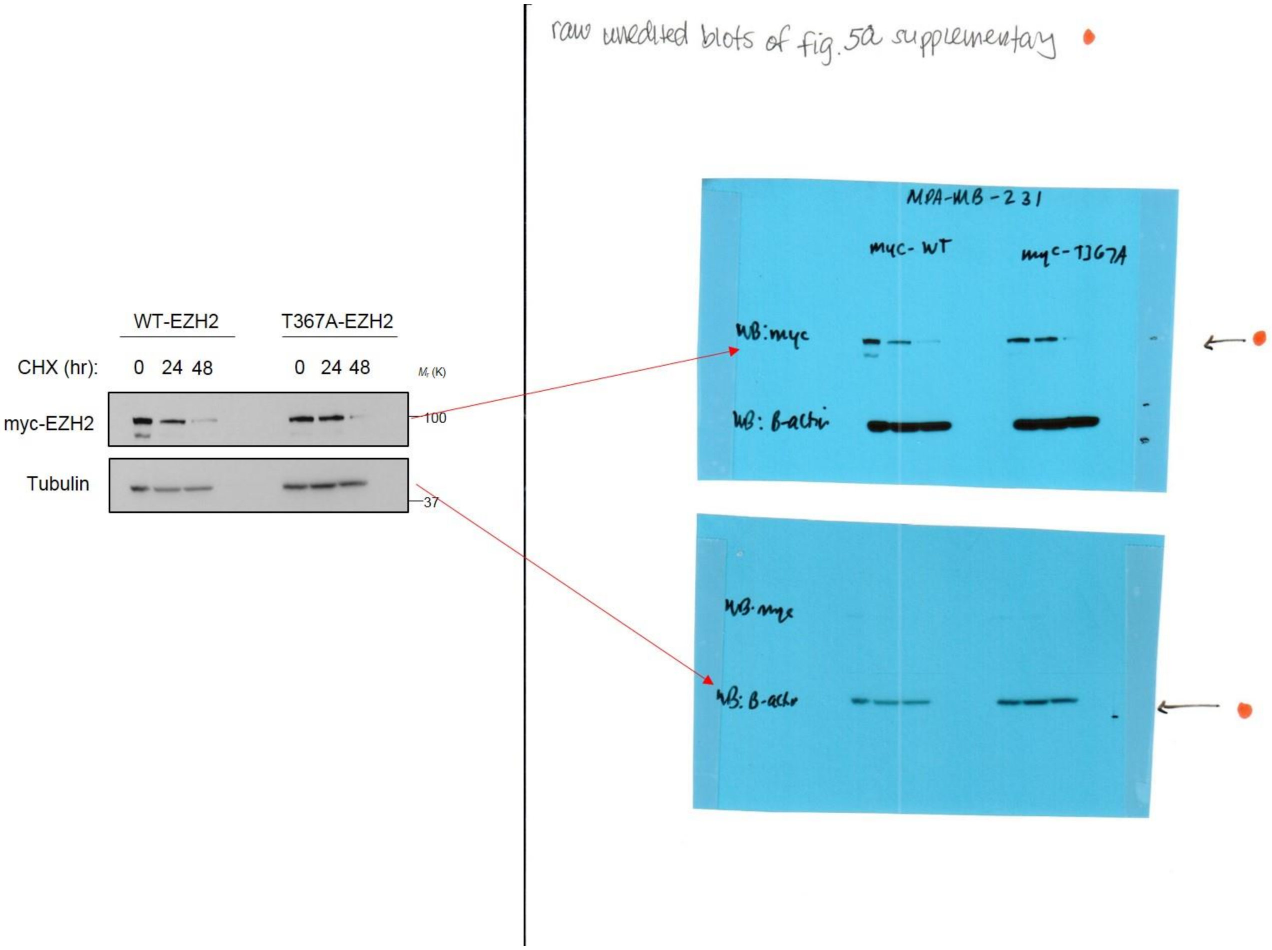

Supplementary Figure 7. Raw/uncropped western blot scans

Supplementary Figure 5b

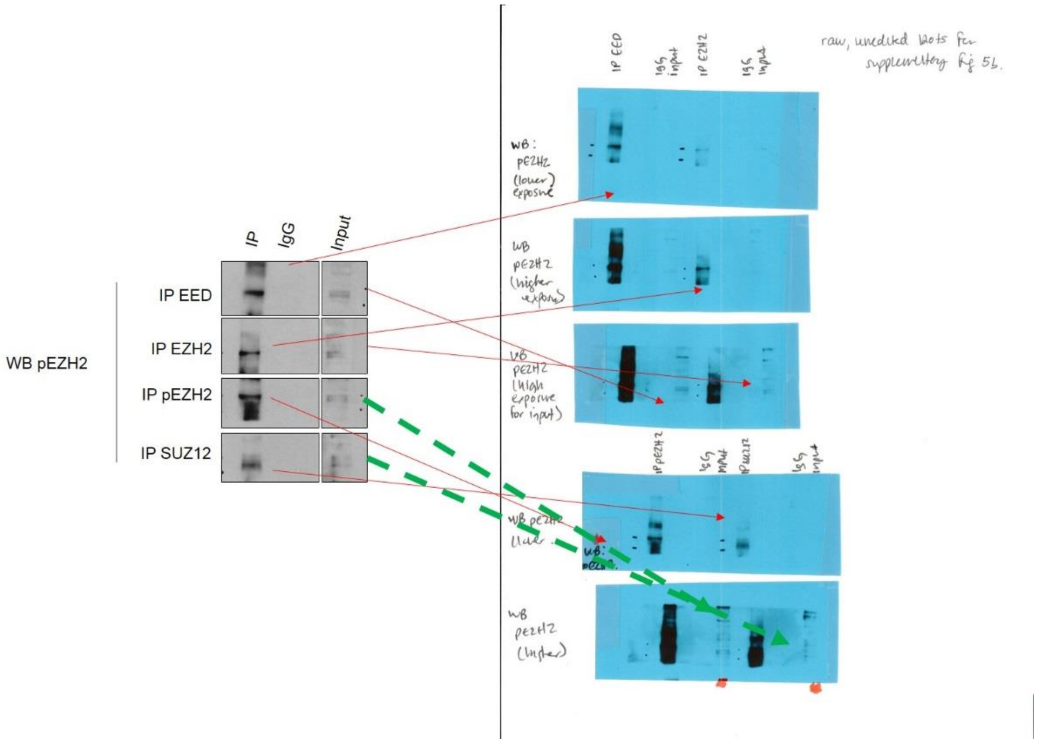

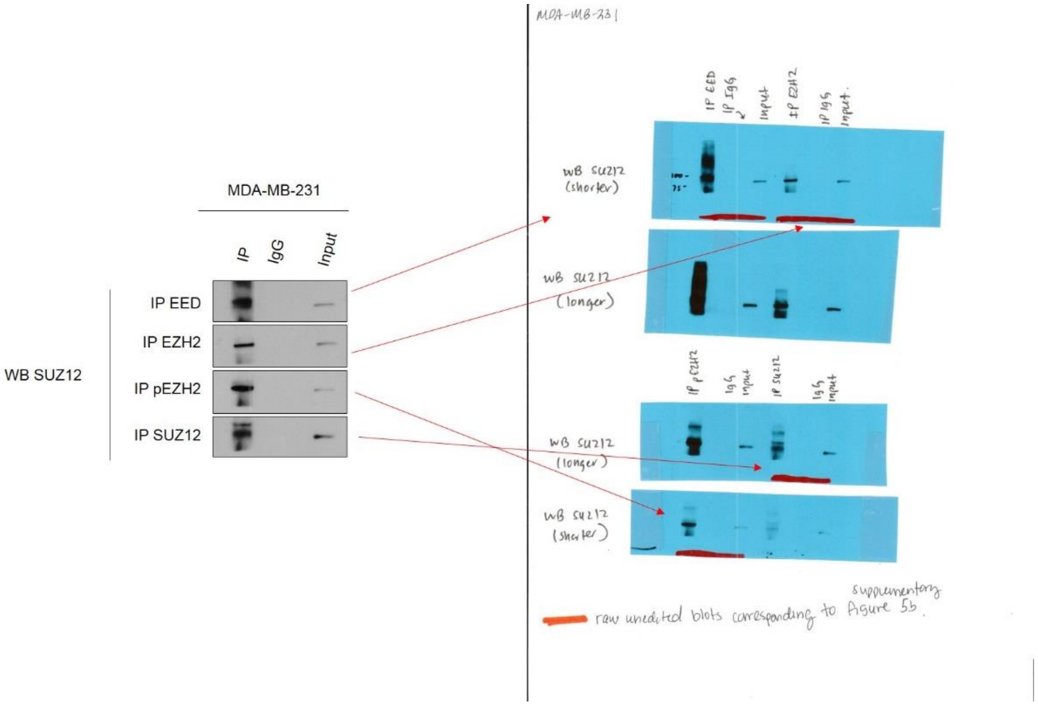

Supplement: Supplementary file 1 — Supplementary Information [file 41467_2018_5078_MOESM1_ESM.pdf]
